# Differential Regulation of Telomeric Complex by *BCR-ABL1* Kinase in Human Cellular Models of Chronic Myeloid Leukemia—From Single Cell Analysis to Next-Generation Sequencing

**DOI:** 10.3390/genes11101145

**Published:** 2020-09-29

**Authors:** Anna Deregowska, Monika Pepek, Katarzyna Pruszczyk, Marcin M. Machnicki, Maciej Wnuk, Tomasz Stoklosa

**Affiliations:** 1 Department of Biotechnology, University of Rzeszow, Pigonia 1, 35-310 Rzeszów, Poland; deregowskaanna@o2.pl; 2 Postgraduate School of Molecular Medicine, Medical University of Warsaw, Trojdena 2a, 02-091 Warsaw, Poland; m.pepek91@gmail.com (M.P.); marmach.marmach@gmail.com (M.M.M.); 3 Department of Immunology, Medical University of Warsaw, Nielubowicza 5, 02-097 Warsaw, Poland; katarzynapruszczyk@gmail.com; 4 Department of Tumor Biology and Genetics, Medical University of Warsaw, Pawinskiego 7, 02-106 Warsaw, Poland

**Keywords:** chronic myeloid leukemia, blast crisis, BCR-ABL1, telomere length, telomerase, shelterin complex, alternative lengthening of telomeres, next-generation sequencing

## Abstract

Telomeres are specialized nucleoprotein complexes, localized at the physical ends of chromosomes, that contribute to the maintenance of genome stability. One of the features of chronic myeloid leukemia (CML) cells is a reduction in telomere length which may result in increased genomic instability and progression of the disease. Aberrant telomere maintenance in CML is not fully understood and other mechanisms such as the alternative lengthening of telomeres (ALT) are involved. In this work, we employed five *BCR-ABL1*-positive cell lines, namely K562, KU-812, LAMA-84, MEG-A2, and MOLM-1, commonly used in the laboratories to study the link between mutation, copy number, and expression of telomere maintenance genes with the expression, copy number, and activity of *BCR-ABL1*. Our results demonstrated that the copy number and expression of *BCR-ABL1* are crucial for telomere lengthening. We observed a correlation between *BCR-ABL1* expression and telomere length as well as shelterins upregulation. Next-generation sequencing revealed pathogenic variants and copy number alterations in major tumor suppressors, such as TP53 and CDKN2A, but not in telomere-associated genes. Taken together, we showed that *BCR-ABL1* kinase expression and activity play a crucial role in the maintenance of telomeres in CML cell lines. Our results may help to validate and properly interpret results obtained by many laboratories employing these in vitro models of CML.

## 1. Introduction

Chronic myeloid leukemia (CML) is a myeloproliferative neoplasm caused by reciprocal translocation t(9;22)(q34;q11), resulting in the formation of the Philadelphia chromosome and *BCR-ABL1* fusion oncogene [1,2]. The hybrid gene *BCR-ABL1* undergoes translation into chimeric protein, which is a constitutively active tyrosine kinase which phosphorylates several target proteins and in effect enables expansion of leukemic stem and progenitor cells. Natural course of the disease progression is characterized by a successive increase in the number of blast cells in the blood and bone marrow and is classified into phases: chronic phase (CP-CML), accelerated phase (AP-CML), and blastic phase (BP-CML), also called blast crisis. Although the introduction of tyrosine kinase inhibitors (TKIs) to the therapy of CML significantly improved the outcome for the great majority of patients, there is still a minor group of patients who develop drug resistance and are at risk of progression. The pathogenesis of BP-CML is still poorly understood and TKIs have limited effectiveness in this phase of the disease [3,4]. One of the features of BP-CML is genomic instability when leukemic stem cells acquire additional genetic changes that may cause drug resistance and lead to disease relapse [5].

Telomere maintenance is crucial for the genomic stability of normal cells, and among several possible mechanisms leading to genomic instability in cancer cells, disrupted telomere maintenance is one of the hallmarks [6]. Telomeres (in eukaryotes termini of chromosomes) are composed of tandem repeats of six base pairs (TTAGGG) and, together with several proteins, named shelterin complex, protect chromosome ends from recognition by DNA repair machinery as double strand breaks (DSBs) and from end to end fusion [7]. In human cancer, telomere shortening and aberrant activation of telomerase is one of the key features of oncogenic transformation [8]. Telomere length is regulated by telomerase complex, which consists of telomerase reverse transcriptase (TERT) and two copies of RNA template (TERC) and also additional proteins stabilizing the complex, such as dyskerin (DKC1) and others (NHP2, NOP10 and GAR1). Telomere maintenance in malignant cells is regulated not only by expression of telomerase complex, but also by various telomere-associated proteins, such as the shelterin complex [9]. The major role of shelterins is to prevent the recognition of telomeres as DNA damage sites. The complex is composed of six proteins: telomeric repeat-binding factors 1 and 2 (TRF1 and TRF2), TRF1-interacting nuclear factor 2 (TIN2), protection of telomeres (POT1), POT1 and TIN2-interacting protein 1 (TPP1), and TRF2-interacting protein 1 (RAP1) [9]. Additionally, other telomeric-associated proteins, such as TEP1 and tankyrase, interact with the shelterin complex. In general, TERT complex and tankyrase are considered as positive regulators of telomere length, while TRF1, TRF2, and POT1 are negative regulators [9].

In CML, telomere attrition has been associated with disease progression [10]. Telomeres are significantly shorter in BP-CML patients’ cells as compared to cells from CP-CML, while the latter are shorter than in cells from healthy donors [11,12]. It has been proposed that telomere shortening can be considered as a prognostic marker in CML [13]. Interestingly, in contrast to many advanced solid tumors, TERT expression is rather downregulated in BP-CML as compared to CP-CML and reduced TERT expression has been attributed to telomere shortening in CML patients [14]. Therefore, other mechanisms than the activation of TERT are possibly involved in dealing with critically short telomeres, especially in BP-CML.

The aim of this study was to investigate expression and activity of genes involved in different mechanisms of telomere maintenance as well as mutational status of the most significant members of the telomerase complex and shelterins, in widely used CML cell lines. Additionally, a possible link between aberrant telomere regulation and genomic instability in BP-CML cells has been examined. We employed five well-established *BCR-ABL1*-positive cell lines, all derived from BP-CML patients, namely K562, KU-812, LAMA-84, MEG-A2, and MOLM-1, and additionally HL-60 as *BCR-ABL1*-negative acute myeloid leukemia cell line as a control.

## 2. Materials and Methods 

### 2.1. Cell Lines 

All human cell lines (HL-60, K562, KU-812, LAMA-84, MEG-A2 and MOLM-1) were obtained from the Deutsche Sammlung von Mikroorganismen und Zellkulturen (DSMZ, Braunschweig, Germany). HL-60, K562, LAMA-84 were cultured in RPMI-1640 medium supplemented with 10% fetal bovine serum (FBS). KU-812, MOLM-1 were cultured in RPMI-1640 medium supplemented with 20% FBS and MEG-A2 were cultured in IMDM medium supplemented with 20% FBS. All media were supplemented with antibiotic solution (100 U/mL penicillin, 0.1 mg/mL streptomycin and 0.25 µg/mL amphotericin B) (all from Life Technologies, Carlsbad, CA, USA). Cells were maintained at 37 °C in 5% CO_2_ in a humidified incubator and were routinely tested for mycoplasma contamination using a PCR-based test.

### 2.2. Telomere Length Analysis and Telomerase Activity

Telomere length was analysed by two different methods, Southern blotting analysis of the genomic DNA and additionally at the level of the single cell by fluorescence in situ hybridization (FISH). The genomic DNA was extracted using the Wizard^®^ Genomic DNA Purification Kit (Promega, Madison, WI, USA) according to the protocol provided by the manufacturer’s recommendations. Average telomere length was measured by Southern blot analysis of terminal restriction fragments (TRF) using the TeloTAGGG telomere length assay kit (Roche, Basel, Switzerland) according to the manufacturer’s instructions as described previously [15]. Mean TRF length for each cell line was estimated on the base of the highest signal intensity peak from TRF due to multiple hybridization of telomeric-specific hybridization probe. Densitometric profile was performed to correspond to bands of DNA marker using ImageJ with gel analysis module. Metaphase spreads and interphase nuclei were obtained from routine 24 h cell cultures according to protocol described in 2.1 material and methods section. To stop cell division the colchicine was added to a final concentration of 0.02 μg/mL and then incubate at 37 °C for a 2 h. After incubation, cells were treated with 75 mM KCl at 37 °C for 15 min and fixed in ethanol and glacial acetic acid (3:1) solution. Hybridization was performed with the Telomere PNA FISH kit/Cy3 (Dako, Glostrup, Denmark) according to the protocol provided by the manufacturer’s recommendation. Fluorescent signals were visualized under the Olympus BX61 with objective 40× and at least 100 interphase nuclei were analysed to determine the telomere lengths in each cell line. Telomere intensities were analysed using TFL-TELO Software, version 2.0, software (British Columbia Cancer Center, Vancouver, Canada). Telomerase activity was measured with a TeloTAGGG Telomerase PCR ELISA kit (Roche, Basel, Switzerland) according to the manufacturer’s instructions.

### 2.3. Fluorescence In Situ Hybridization (FISH)

FISH was performed using probes for: *BCR-ABL1* t(9;22) Fusion (KBI-10005, Kreatech, Amsterdam, The Netherlands), *JAK2* (9p24) Break (KBI-10012, Kreatech, Amsterdam, Netherlands), *PDGFRB* (5q32) Break (KBI-10004, Kreatech, Amsterdam, The Netherlands), *TERT* (5p15) (KBI-40113, Kreatech, Amsterdam, The Netherlands) or *TERC* (3q26)/3q11 (KBI-10110, Kreatech, Amsterdam, The Netherlands). For FISH experiments, a standard protocol was used. In brief, the cells were fixed with ethanol and glacial acetic acid (3:1) solution and treated with RNAse (100 µg/mL) in 2 × SSC buffer for 1 h at 37 °C in humidity chamber. After washing, first in PBS and then in PBS with 50 mM MgCl_2_, the slides were dehydrated in ethanol series. FISH probe was denatured together with the slide at 80 °C for 7 min and hybridized overnight in the dark at 37 °C in humidity chamber. The slides were washed, first at 72 °C and then at RT (0.4% Igepal in 2 × SSC and 2% Igepal in 2 × SSC, respectively). For DNA visualization, the slides were counterstained with a drop of mounting medium with 4′,6′-diamino-2-phenylindole counterstain (ProLong™ Diamond Antifade Mountant with DAPI, Invitrogen™ (Carlsbad, CA, USA). Fluorescent signals were visualized under the Olympus BX61 and MetaSystem Isis software (Altlußheim, Germany) with objective 40×. 100 interphase cells were examined. The signal pattern was analysed according to the manufacturer’s instructions.

### 2.4. Western Blotting

Cell pellets were lysed with ice-cold RIPA buffer (50 mM Tris–HCl pH 7.5, 150 mM NaCl, 1 mM EDTA, 1% NP-40, 0.1% SDS, 0.5% sodium deoxycholate, 1 mM PMSF and 1× complete protease inhibitors, Roche, Basel, Switzerland) and were boiled in 2 × Laemmli’s sample buffer. Lysates were separated by 10% SDS-PAGE at 120 volts for 60 min (Bio-Rad, Hercules, CA, USA) and transfer by electroblotting (Bio-Rad, Hercules, CA, USA) onto polivinylidene difluoride (PVDF) membrane (Merck Millipore, Burlington, MA, USA) at 4 °C at a constant voltage of 100 V for 100 min in a Towbin buffer (25 mM Tris, 192 mM glycine, 0.1% SDS and 20% ethanol). Membrane was blocked in TBS-T (Tris-buffered saline-Tween20) buffer containing 2% BSA for 60 min at room temperature and then incubated overnight at 4 °C in 1% BSA in TBS-T containing primary antibody: anti-phospho-CRKL (Tyr207) (1:500, #3181), anti-CRKL (1:500, #3182), anti-POT1 (1:1000, MAB5299), anti-RAP1 (1:1000, PA5-35137), anti-TERT (1:1000,ab32020), anti-TRF1 (1:1000, NB100-1701), anti-TRF2 (1:1000, P5-19426,), anti-HSP70 (1:1000, PA5-14521), anti-HSP90 (1:1000, MA1-10373) or anti-β-Actin (1:40,000, A3854). After three washes for 5 min each in TBS-T, they were incubated for 60 min at room temperature with secondary antibody (#7076S or #7074S) diluted 1:2000 in 1% BSA in TBS-T, and washed as described above. Antibodies were acquired from Cell Signaling Technology (Danvers, MA, USA), R&D Systems (Minneapolis, MN, USA), Abcam (Cambridge, United Kingdom), Novus Biologicals (Centennial, CO, USA) and Invitrogen™ (Carlsbad, CA, USA). The chemiluminescence signals were detected with an ECL Prime Western blotting Detection Reagent (GE Healthcare, Chicago, IL, USA) and a G:BOX imaging system (Syngene, Bangalore, India). Densitometry analysis was conducted using ImageJ software (https://imagej.nih.gov/ij/) (U. S. National Institutes of Health, Bethesda, MD, USA).

### 2.5. Immunofluorescence

For promyelocytic leukemia protein (PML) and TRF2 co-localization, interphase nuclei were used. The cells were fixed with 3.7% formaldehyde in PBS for 20 min in 1.5 mL tube. After 3 times washing with PBS, the cells were transferred on Polysine Slides (Thermo Scientific™ Shandon™ Polysine Slides, Thermo Scientific, Waltham, MA, USA) and permeabilized with 0.3% Triton X-100 in PBS for 5 min at RT and next blocked with 1% BSA in PBS-T (PBS-Tween20) at RT for 30 min. After washing with PBS-T, the cells were incubated with antibodies: anti-PML (1:200, ab96051) and anti-TRF2 (1:100, ab108997) (Abcam, Cambridge, UK) overnight at 4 °C. After incubation, cells were washed twice with PBS and incubated with secondary antibodies: FITC-conjugated and TexasRed-conjugated, respectively (all at 1:000, F2761, T6390) (Life Technologies, Carlsbad, CA, USA) for 1 h at RT in the dark. Cells were then washed three times with PBS and nuclei were stained with DAPI. Images were taken using an Olympus BX61 fluorescent microscope (Shinjuku, Japan) with objective 20×. To analyze co-localization PML/TRF2, ImageJ software http://rsbweb.nih.gov/ij/ with JACoP plugin was used [16]. The Pearson’s coefficient was used to calculating a set of co-localization indicators.

### 2.6. DNA Damage and DNA Damage Response

DNA double strand breaks (DSBs) were analyzed using neutral comet assay upon stimulation with DSBs inducer: 10 µM etoposide for 24 h, as described previously [17]. The cells were cultured according to the protocol described in the Material and Methods section. The all cell cultures including positive control with etoposide treatment as well as negative control without etoposide were performed in three independent experiments after 24 h cell culture. The percentage of tail DNA was used as a parameter of DNA damage. Images were taken using an Olympus BX61 fluorescent microscope with objective 20×. Comets (*n* = 100) were analyzed using CASP, an open-source software tool 1.2.3beta2 version (http://casplab.com) (Wroclaw, Poland). The activation of ATM and H2AX was measured using flow cytometry and Muse™ Multi-Color DNA Damage kit (Merck Millipore, Burlington, MA, USA) as described elsewhere [18].

### 2.7. Detection of Micronuclei (MN)

The slides were fixed with ethanol and glacial acetic acid (3:1) solution and were stained with DAPI. The results were expressed as the number of MN-positive cells per 100 nuclei (%).

### 2.8. Real-Time PCR

The reverse transcription quantitative PCR (RT-qPCR) was used to evaluate expression level of the following genes: *BCR-ABL1*, *TRF1*, *TRF2*, *TERT*, *TERC*, *RAP1* and *POT1*. RNA extraction (TriPure Isolation Reagent, Sigma-Aldrich, Saint Louis, MO, USA) followed by reverse transcription (Transcriptor First Strand cDNA Synthesis Kit, Roche, Basel, Switzerland) were performed according to the manufacturer’s manuals. RT-qPCR was carried out in LightCycler™ 480 instrument (Roche, Basel, Switzerland) using LightCycler^®^ 480 Probes Master and Universal Probe Library (Roche, Basel, Switzerland) in a final volume of 10 µL. *β2M* and *GUSB* were used as reference genes. 

### 2.9. Next-Generation/Sanger Sequencing

Mutational analysis of coding sequences of genes associated with haematological malignancies as well as typical tumor suppressors and oncogenes was carried out using targeted enrichment by one of our two custom-made gene panels (targeting genes relevant to human malignancies). Lists of sequenced genes are presented in Supplementary Table S1. Genomic DNA was isolated using Gentra Puregene Cell Kit (Qiagen, Hilden, Germany) according to the manufacturer’s protocol. DNA libraries were prepared from 0.5 μg DNA using Kapa Library Preparation Kit for Illumina (Roche, Basel, Switzerland), multiplexed with others before solution-based capture (Roche NimbleGen, Basel, Switzerland). The capture protocol was performed according to the manufacturer’s recommendations. Briefly, 1 μg of pooled DNA was hybridized for 72 h with the probes, then after washing steps, the captured DNA was amplified, purified, qualified using Bioanalyzer (Agilent Technologies, Santa Clara, CA, USA) and quantified using Qubit (Life Technologies, Carlsbad, CA, USA). Cell lines were sequenced on Illumina HiSeq 1500 (Illumina, San Diego, CA, USA) to achieve mean depth of coverage in range 63.9–107.6× followed by GATK 4.0.1.1-based variant discovery [19]. Variant discovery comprised of quality control of raw fastq, adapter trimming and low quality reads removal using Trimmomatic [20], read mapping to hg19 genome using BWA-MEM 0.7.15-r1140 [21], duplication removal, local realignment and quality recalibration using GATK and Picard and variant calling using HaplotypeCaller. Variants more common than 1% in public (1000 genomes, NHLBI ESP, gnomAD) databases as well as variants more common than 5% in our internal database were removed from the analysis. To identify functional consequences of detected variants five bioinformatics predictors were used: CADD, PolyPhen2, SIFT, FATHMM and Mutation Taster. Copy-number calling was performed by sequencing coverage analysis using CNVkit v0.9.5 [22]. Non-leukemic blood samples and leukemic blood samples verified as devoid of detectable copy number variations were used to create coverage references for panels 1 and 2, respectively. CNVkit was run with default settings, except that bin size was limited to 400 bp to increase resolution for both panels. Additionally, off-target coverage was not used in CNV calling for panel 2. CNVkit built-in function center-at was used to correct log2 coverage ratio values in samples with copy-number-neutral regions incorrectly placed under 0 (for KU-812 and MEG-A2, Supplementary Figures S8 and S9, Supplementary Table S2). 

*TERT* promoter hotspot mutations (C228T and C250T) were analysed by NGS, but due to low coverage of the promotor region, also additionally confirmed by Sanger sequencing as described [23], with modifications. Briefly, primer sequences were as follows: TERT-F: AGGAGGCTCTTTGGAGCAAG, TERT-R: CTCTGAACTCTGTGCTGACCA. PCR was performed using KAPA HiFi HotStart ReadyMix PCR Kit (Roche, Basel, Switzerland) with the program 3 min at 95 °C followed by 35 cycles of 20 s at 98 °C, 10 s at 71 °C and 10 s at 72 °C and final extension at 72 °C for 5 min. Before sequencing on a ABI3730 Genetic Analyzer (Applied Biosystems, Foster City, CA, USA), the PCR products were purified using Agencourt AMPure XP beads (Beckman Coulter, Brea, CA, USA) and labelled with BigDye Terminator v.3.1 (Applied Biosystems, Foster City, CA, USA).

### 2.10. Statistical Analysis

Statistical significance was assessed using GraphPad Prism 6.07 (GraphPad Software, Inc., La Jolla, CA, USA) by one-way ANOVA and with the Tukey’s multiple comparison test. Differences between control conditions versus etoposide treatment were assessed with the one-way ANOVA with the Dunnett’s multiple comparison test. The correlation analysis was performed using linear correlation (Pearson r) test. *p*-Values of less than 0.05 were considered significant.

## 3. Results

### 3.1. Cytogenetic and Molecular Analysis of BCR-ABL1-Positive Cell Lines Reveal Complex Karyotype and Different Expression of BCR-ABL1 Oncogene

**Figure 1:**
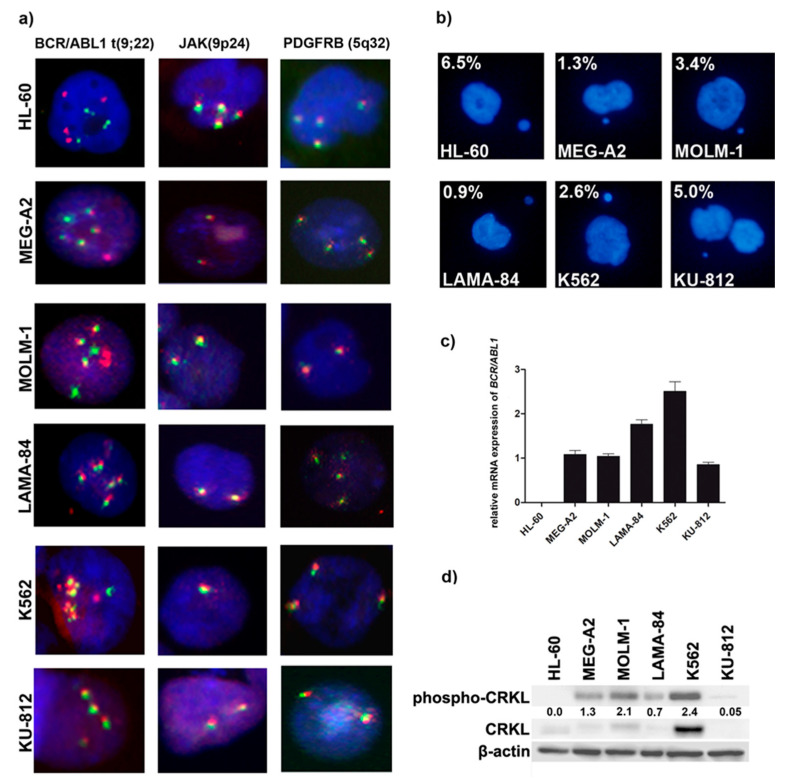
Cytogenetic and molecular features of *BCR/ABL1*-positive cell lines. (**a**) Representative images of fluorescence in situ hybridization with *BCR-ABL1* t(9;22) fusion probe, *JAK2* (9p24) break probe and *PDGFRB* (5q32) break probe. Fluorescent signals were visualized under the Olympus BX61 and MetaSystem Isis software with objective 40×; (**b**) Representative images and percentages of micronuclei (MN) positive cells expressed as %; (**c**) RT-qPCR analysis of *BCR/ABL1* normalized to *β2M* and *GUSB*; (**d**) Assessment of BCR/ABL1 kinase activity, as determined by Western blotting analysis of phospho-CRKL. For the loading control, β-actin was used.

FISH was employed to characterize the most commonly described chromosomal aberrations associated with CML in addition to the Philadelphia chromosome (Figure 1a). The gene copy numbers of *BCR-ABL1*, *JAK2* and *PDGFRB* are presented as representative microphotographs of nuclei after hybridization with *BCR-ABL1* t(9;22) fusion probe, *JAK2* (9p24) break probe and *PDGFRB* (5q32) break probe (Figure 1a, Supplementary Figure S1). The FISH analysis revealed that all CML cell lines have a balanced t(9;22) translocation, based on a fusion of the 3′ *ABL1* region at 9q34 with the 5′ *BCR* region at 22q11, while HL-60 was Philadelphia chromosome-negative, as expected (Figure 1a). The LAMA-84, MOLM-1, and MEG-2A cell lines had at least three Philadelphia chromosomes. The KU-812 cell line contains at least one Philadelphia chromosome while most nuclei of K562 had two (observed in ~34% cells) or three (observed in ~63% cells) multiple foci as typical fusion signals as consequences of multiamplification of *BCR-ABL1*. We have noted that applied diagnostic FISH of *BCR-ABL1* in cell line with multiple foci of BCR-ABL1 is limited due to overlapping signals, therefore we have used NGS data for additional, confirmatory CNV analysis. We confirmed in CNV analysis of NGS data high-level amplification of *BCR-ABL1* gene in K562 cell line as indicated by presence of more than 10 copies of *ABL1* and *BCR* genes (Supplementary Table S2). In FISH, HL-60 cells showed 4 signals for *ABL1* as well as *BCR* gene, which is explained by the tetraploidy of these cells (Figure 1a). 

Additionally, because Janus kinase 2 (JAK2) regulates BCR-ABL1 signaling in chronic myeloid leukemia, we decided to analyze the copy number of *JAK2* gene. FISH analysis did not show translocations involving *JAK2* in any of analyzed cell lines (Figure 1a). However, we observed variability in *JAK2* gene copy number between different cell lines and between single cells within the same cell line (additionally confirmed with CNV analysis by NGS (Supplementary Table S2)). The highest number of *JAK2* gene copies were present in HL-60 cells, again, due to tetraploidy with majority of CML cell lines having two *JAK2* gene signals, and most of K562 cells with only one copy of *JAK2* (Figure 1a). We then analyzed *PDGFRB* loci that might be associated with several translocation partners, the best known is the *ETV6* gene on 12p13, causing a t(5;12) translocation that is observed in patients with other myeloproliferative neoplasms. Moreover, we decided to analyze the copy number of *PDGFRB* gene because promotor of *PDGFRB* gene is controlled by the telomeric protein TRF2 [24]. In general, none of cell line had breaks in *PDGFRB* loci (Figure 1a), but we observed some extent of variability in *PDGFRB* gene copy number between cell lines as well as single cells of the same line (from 2 signals in KU-812 to 4 signals in MEG-A2). 

Micronuclei are markers of genomic instability, specifically, chromosomal instability. Analysis of micronuclei production between all cell lines showed that HL-60 (*BCR-ABL1*-negative cell line) is the most prone to micronuclei formation (Figure 1b). Among CML cell lines, KU-812 cells showed the highest frequency of micronuclei, while the lowest frequency was observed in MEG-2A and LAMA-84 cells (Figure 1b). To further characterize CML cell lines, we also assessed *BCR-ABL1* expression level (Figure 1c) and kinase activity, as determined by the phosphorylation status of CRKL, adaptor protein targeted by BCR-ABL1 kinase activity (Figure 1d). The highest level of *BCR-ABL1* transcription was observed in K562 cells. In contrast, KU-812 cells showed more than 2-fold lower *BCR-ABL1* transcript level as compared to K562 and LAMA-84 cells (Figure 1c). 

Additionally, we analyzed the phosphorylation of adaptor protein CRKL, routinely used to assess BCR-ABL1 kinase activity (Figure 1d). High expression of *BCR-ABL1* transcript in K562 was associated with the highest level of kinase activity, this confirms the amplification of *BCR-ABL1* in this cell line by FISH and NGS and also amplification of *CRKL* in this cell line (Supplementary Figure S5). However, in the remaining cell lines *BCR-ABL1* expression was not clearly correlated with the kinase activity. According to the activity of BCR-ABL1 kinase, CML cell lines can be ranked as follows: K562 > MOLM-1 > MEG-2A > LAMA-84 > KU-812 cells (Figure 1d).

### 3.2. Different Telomere Length in BP-CML Cells Is Associated both with BCR-ABL1 Expression and Kinase Activity

**Figure 2:**
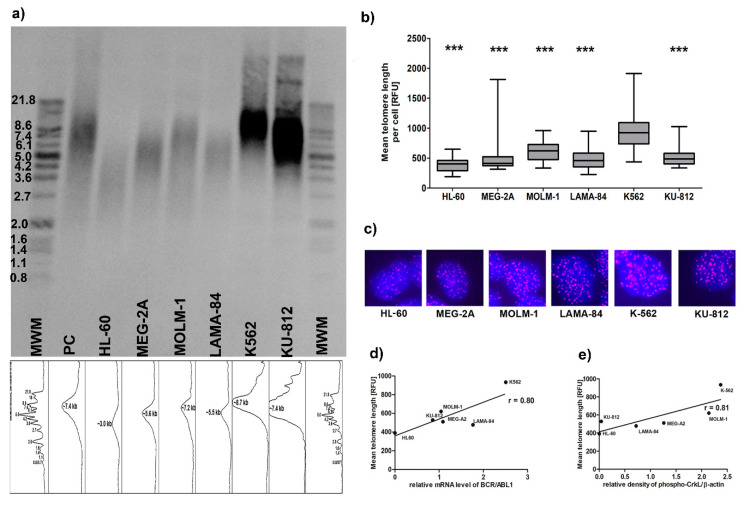
Telomere length dynamics in *BCR/ABL1*-positive cell lines. Global picture of telomere length: (**a**) Southern blot analysis of telomere length, MWM- molecular weight marker, PC-positive control; Mean TRF length for each cell line was estimated on the base of the highest signal intensity peak from TRF due to multiple hybridization of telomeric-specific hybridization probe. Densitometric profile was performed to correspond to bands of DNA marker using ImageJ with gel analysis module. (**b**) telomere length in a single cell: fluorescence in situ hybridization (FISH) with PNA technique. The bars indicate SD, *n* = 100, *** *p* < 0.001 compared to the K562 (ANOVA and Tukey’s a posteriori test); (**c**) Representative images of PNA. Fluorescent signals were visualized under the Olympus BX61 with objective 40×; (**d**,**e**) Telomere length positively correlates with *BCR/ABL1* expression and activity: (**d**) Pearson’s (r) correlations of mean telomere length and *BCR/ABL1* expression and (**e**) activity of BCR/ABL1 kinase expressed as relative density of phospho-CRKL normalized to β-actin.

Since the loss of telomeres in cancer cells may result in increased genomic instability, we checked if there is a link between telomere length and *BCR-ABL1* expression and/or kinase activity (Figure 2). We found that CML cell lines exhibit significant differences in telomere length, notably all having longer telomeres than HL-60 cells. The longest telomeres were observed in K562 cells according to results obtained using TeloTAGGG telomere length assay (Figure 2a) as well as FISH-PNA analysis (Figure 2b,c, Supplementary Figure S2). Moreover, we revealed that telomere length was strongly correlated with *BCR-ABL1* expression (Figure 2d) and BCR-ABL1 kinase activity (Figure 2e).

### 3.3. Telomerase Expression and Activity Is Not a Critical Factor in Telomere Maintenance of BP-CML Cell Lines

TERT complex is required for the addition of telomeric repeats to the termini of eukaryotic chromosomes, therefore we decided to evaluate if telomere length could be altered by different expression and activity of telomerase complex in studied cells. *TERT* and *TERC* gene copy number and gene expression were analyzed as well as TERT protein levels and enzymatic activity in all cell lines.

**Figure 3:**
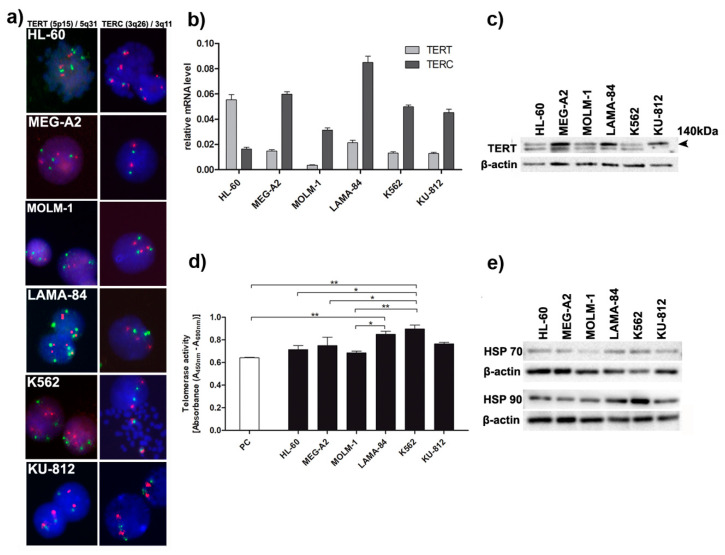
Telomere length is associated with enzymatic activity of telomerase and with expression/gene copy number of *TERT/TERC* in *BCR/ABL1*-positive cell lines. (**a**) Representative images of nuclei and/or metaphase plates with *TERT/TERC* probe. Nuclei were counterstained by DAPI. Fluorescent signals were visualized under the Olympus BX61 and MetaSystem Isis software with objective 40×; (**b**) RT-qPCR analysis of *TERT/TERC* normalized to *β2M* and *GUSB* expression; (**c**) Western blot analysis of TERT protein levels. For the loading control, the antibody against β-actin was used; (**d**) PCR-ELISA measurement of telomerase activity. The bars indicate SD, *n* = 2, * *p* < 0.05, ** *p* < 0.01 (ANOVA and Tukey’s a posteriori test); (**e**) Western blotting analysis of HSP70 and HSP90. For the loading control, β-actin was used.

According to the FISH analysis, most CML cell lines revealed at least two fluorescent signals for *TERT* and *TERC* genes with only KU-812 cells presented in some of the nuclei a single copy of *TERT* gene (Figure 3a), which also was confirmed by CNV analysis of NGS data for most of the cell lines, except KU-812 (Supplementary Table S2).

RT-qPCR analysis of *TERT/TERC* normalized to *β2M* and *GUSB* expression showed higher expression of *TERC* gene in all CML cell lines in comparison to HL-60 cells (Figure 3b). In contrast, the *TERT* gene expression was higher in HL-60 cells compared to CML cell lines (Figure 3b). The analysis of TERT expression at protein level showed that the highest expression of this protein has MEG-2A, LAMA-84, and KU-812 cells, respectively (Figure 3c). Although, the levels of *TERT* and *TERC* expression did not correspond to the enzymatic activity of telomerase, the highest telomerase activity was observed in K562 cells (Figure 3d). Therefore, we decided to evaluate the levels of HSP70 and HSP90, the chaperone proteins, essential for telomerase stability and activity (Figure 3e). The level of HSP70 as well as HSP90 expression was associated with telomerase activity in CML cell lines, with K562 cells having the highest expression of both chaperones.

### 3.4. Shelterin Complex Is Activated in BP-CML Cells

**Figure 4:**
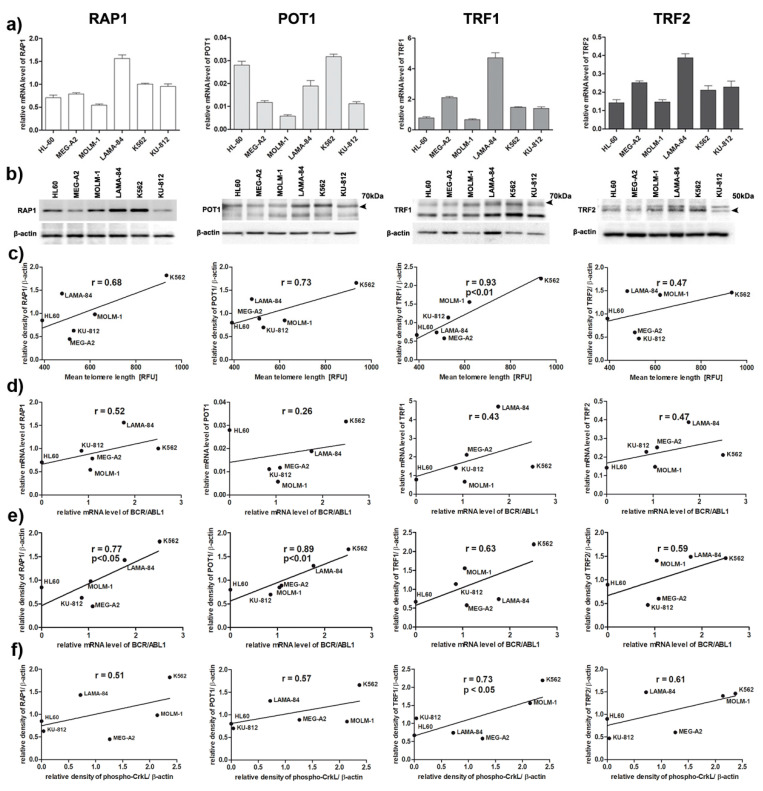
Role of selected shelterin proteins in telomere maintenance in *BCR/ABL1*-positive cell lines. (**a**) RT-qPCR analysis of shelterin genes. Relative quantification of each gene expression level was normalized according to the gene expression *β2M* and *GUSB*; (**b**) Western blotting analysis of RAP1, POT1, TRF1 and TRF2. β-Actin served as a loading control; (**c**–**f**) Pearson’s (r) correlations of: (**c**) mean telomere length and shelterin proteins level; (**d**) *BCR/ABL1* expression and *RAP1*, *POT1*, *TRF1*, *TRF2* genes expression; (**e**) *BCR/ABL1* expression and shelterin proteins level; (**f**) activity of BCR/ABL1 kinase and protein level of RAP1, POT1, TRF1, TRF2.

Shelterin complex plays a crucial role not only in telomere protection and maintenance but also in telomerase activity. Therefore, we tested if the expression pattern at the mRNA and protein level of the selected shelterin complex members, namely: *RAP1, POT1, TRF1* and *TRF2* can explain differences in telomere dynamics in *BCR-ABL1*-positive cell lines (Figure 4a,b). We correlated the obtained results with telomere length, *BCR-ABL1* expression, and activity of BCR-ABL1 as measured by phospho-CRKL (Figure 4c–f).

Increased expression at protein level in all analyzed proteins was positively correlated with longer telomeres, but only for TRF1 was statistically significant (Figure 4c). Additionally, expression of *RAP1*, *POT1*, *TRF1* and *TRF2* at mRNA and protein level was positively correlated with the levels of expression of *BCR-ABL1*, reaching statistical significance for RAP1 and POT1 at the protein level (Figure 4d,e). There was a significant positive correlation between TRF1 protein and the activity of BCR-ABL1 (Figure 4f). No other significant correlations were observed. 

Aberrant regulation of the telomere length may be caused by mutations in telomerase complex and/or shelterin genes [25]. In order to verify whether observed differences in the expression and/or activity are caused by genetic aberrations we analyzed NGS data and detected several aberrations, including point mutations, small indels, and large deletions in major tumor suppressors, such as *TP53*, *CDKN2A*, and *ATM* (Table 1). 

Additionally, by using our custom gene panels, covering from almost 200 to 1000 genes involved in human malignancies, we were able to check the mutational status of other genes frequently mutated in human malignancies (Table 1) as well as identify copy number variations (CNVs) (Supplementary Table S2). We confirmed high mutational burden as reflected by *TP53* loss either by mutation or del17p in all cell lines and deletion of *CDKN2A*, and mutations in *ATM* in most of the cell lines. We also detected mutations in *ASXL1* gene as typical frameshift/stop-gain variants described in myeloid malignancies. 

By contrast, we did not detect any variants in telomere-associated genes, except single variant p.R951Q in *TERT* in K562 cell line, which was additionally confirmed by Sanger sequencing (Supplementary Figure S3). This variant has been described already in glioblastoma [26], and in our previous study focused on K562 cells [27]. Mutation in *TERT* promoter were shown to play major role in the activation of telomerase in several tumors, such as melanoma, glioblastoma, head and neck cancer and other solid tumors [28,29]. *TERT* promoter sequence, although included in our targeted enrichment, typically yield only limited number of reads. Therefore, we confirmed the status of the two most common hotspots in the promotor region [29], with Sanger sequencing with negative results in all studied cell lines (Supplementary Figure S4). Additionally, scatter plots with CNV data are presented in Supplementary Figures S5–S10.

Since PNA single cell analysis, revealed a high level of heterogeneity in telomere length within one cell, we performed immunofluorescence to analyze the formation of specialized alternative lengthening of telomeres-associated promyelocytic leukemia (PML) nuclear bodies (APBs), commonly used as a marker of ALT. We evaluated co-localization of APBs/TRF2 using ImageJ software http://rsbweb.nih.gov/ij/ with JACoP plugin, but our analysis showed week co-localization between TRF2 and APBs in any cell line, except in K562 cells (Supplementary Figure S11). This suggests marked activation of ALT in K562 cells as compared to other cell lines.

### 3.5. Response to DNA Double-Strand Breaks Differs in BP-CML Cell Lines

**Figure 5:**
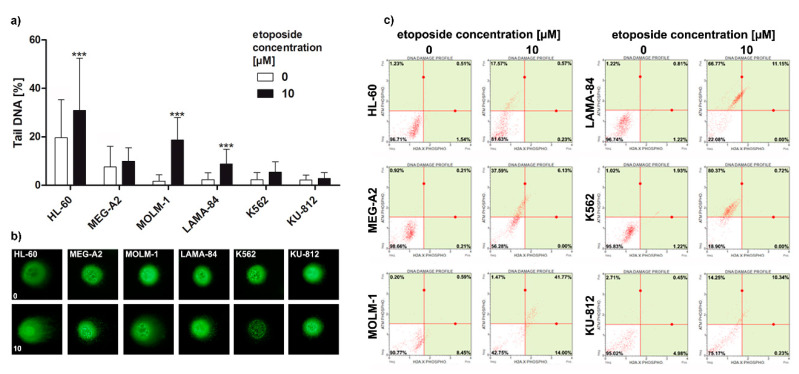
DNA damage response to double strand breaks (DSBs) in *BCR/ABL1*-positive cell lines. (**a**) Neutral comet assay analysis of DSBs. As an DSBs inducer, 24 h treatment with 10 µM etoposide was used. DNA damage marker, the % tail DNA was used. The bars indicate SD, *n* = 100, *** *p* < 0.001, compared to the control (ANOVA and Dunnett’s a posteriori test); (**b**) The typical images are shown. DNA was visualized using YOYO-1 staining. Images were taken using an Olympus BX61 fluorescent microscope with objective 20×; (**c**) phospho-ATM and phospho-H2AX were measured after 24 h treatment with etoposide using flow cytometry and Muse™ Multi-Color DNA Damage Kit.

DNA damage response (DDR) to DSBs in *BCR-ABL1*-positive cell lines was evaluated by neutral comet assay as well as typical markers of DDR (increased phosphorylation of ATM and H2AX). Generally, HL-60 cells were more prone to DNA DSB as compared to CML cell lines. The 10 µM etoposide moderately intensified DNA damage in MOLM-1 and LAMA-84 cells as compared to control cells (Figure 5a,b). 10 µM etoposide treatment promoted DNA damage response, i.e., ATM activation in almost all cell lines examined, however the simultaneous phosphorylation of ATM and H2AX, as markers of DNA damage, were observed with K562 having the lowest and MOLM-1 the highest level of phosphorylation these two proteins among *BCR-ABL1*-positive cell lines (Figure 5c).

## 4. Discussion

According to the current consensus, average telomere length in leukemic cells of CML patients is shorter as compared to white blood cells of age-matched healthy individuals [10,11,12]. Furthermore, telomere shortening was linked to disease progression from chronic to blastic phase of CML, with a shortening rate approximately 10 times higher than in normal controls [13]. Patients with a high-risk Hasford score at diagnosis exhibited significantly greater telomere loss than patients with a low-risk score, while patients with intermediate risk showed an intermediate telomere loss rate [13]. Telomere shortening is more pronounced in leukemic stem cells as compared to normal hematopoietic stem cell compartment at diagnosis as described by Bouillon et al. [30]. It has also been shown that accelerated shortening of telomeres in CML cells can be accompanied by the “telomere-associated secretory phenotype”, driven by RAP1, but not by BCR-ABL1 kinase, however mechanistic explanation of this phenomenon is not complete [31]. Other studies showed that chromosome ends may be unchanged or even elongated in some CML patients. Longer telomeres of chromosome arms have been reported in CML patients compared with healthy donors. Long telomeres may contribute to the cell proliferation during clonal selection in the early stage of CML ontogenesis [13]. This phenomenon can be explained by the mechanisms of alternative lengthening of telomeres (ALT). It has been proposed that ALT plays an important role in early phases of CP-CML, but not in BP-CML [32]. Since it has been postulated that telomerase activity increases with CML progression, the dominating telomere maintenance mechanism might undergo transition from ALT to telomerase-dependent. Therefore, both the fate of the telomeres and the origin of telomere shortening in CML cells are not fully understood.

In the current work, we employed five *BCR-ABL1*-positive cell lines most commonly used in the laboratories for the studies on CML molecular pathogenesis and to test new therapeutic approaches. As a control, we used HL-60 cell line, a *BCR-ABL1*-negative acute leukemia, since normal healthy CD34+ bone marrow cells are not considered as a well-suited control cells for BP-CML cell lines harboring several genetical abnormalities. Our results show that telomeres in cell lines derived from acute phases of CML have different lengths, but all are significantly longer than *BCR-ABL1*-negative HL-60 cells. However, between the CML cell lines telomere length positively correlates with both *BCR-ABL1* expression and activity. K562, which had longest telomeres, demonstrated high level of amplification of *BCR-ABL1* detected at the single cell level by FISH and confirmed by CNV analysis form NGS data and also had highest activity of BCR-ABL1 kinase (Figure 1 and Figure 2 and Supplementary Table S2). FISH analysis was applied to other genes known to be dysregulated in myeloid malignancies such as *JAK2* and *PDGFRB*. *JAK2* gene is a known activator of telomerase and JAK2 inhibitors reduced the telomerase activity [33]. The study of *PDGFRB* gene is of importance because TRF2 regulates expression of *PDGFRB* gene by binding and activating the *PDGFRB* promoter as an example an extra-telomeric function of TRF2 [24]. Although we have found that the copy number of *JAK2* differed between cell lines, it seems irrelevant to telomere maintenance. Similarly, the higher number of *PDGFRB* gene copies also was not associated with longer telomeres or with higher expression of TRF2. The major enzyme required for the addition of telomeric repeats to the termini of eukaryotic chromosomes is telomerase. Human telomerase consists of a human telomerase RNA component (*TERC*), a human telomerase catalytic subunit (TERT) and TEP1, a telomerase associated protein. Previous studies showed that, in CML cells, the expression of *TERT* may be downregulated [14]. Our results showed that although the expression levels of *TERT* and *TERC* gene were generally low in all studied cell lines, K562 cells again demonstrated highest enzymatic activity of telomerase, as compared to other cell lines and highest protein level of both chaperones HSP70 and HSP90 necessary for telomerase activity (Figure 3). 

We have already characterized the global genomic landscape of K562 cell line employing targeted enrichment and next-generation sequencing [27]. In this work, we detected several pathogenic variants, which are described already (such as in *TP53*), but also several new pathogenic variants not previously described in DNA repair genes (such as *BRCA1* or *MLH1*). This may contribute to the aggressive phenotype and genomic instability of this cell line. In the current work, we applied FISH analysis at the level of the single cell and targeted sequencing of several genes involved in human cancer by NGS for the whole panel of cell lines to characterize complex genetic aberrations underlying observed differences in telomere maintenance and telomeric complex genes (Table 1). We did not find any significant defects in the relevant genes involved in telomere maintenance except one pathogenic variant in *TERT* gene in K562 cells (Table 1, Supplementary Figure S3), which we already published [27]. However, we observed inactivation of the major tumor suppressors function, including *TP53* gene (by chromosomal deletion or combination of pathogenic mutation and second allele loss) in all studied cell lines as well as in *CDKNA2* gene encoding p16^INK4a^. The analysis of genetic status of *TP53* and *CDKN2A* is crucial for cellular telomere-damage response pathway in many cells. It is well known that p16^INK4a^ contributes to the p53-independent response to telomere damage and depends from genetic status of p16^INK4a^ and p53, thus, two distinct pathways could be activated [34]. Moreover, it can be postulated that the elongation of telomeres is associated with the loss of p53 and p16^INK4a^ function [35]. This may explain how the cells escape from telomere-mediated cell senescence and/or apoptosis in the BP-CML cell lines, since inactivation of *TP53* was already implicated as a responsible mechanism [36]. 

Analysis of the expression of selected genes, crucial components the telomeric complex, such as shelterins, revealed that BCR-ABL1 kinase expression and activity may upregulate the level of RAP1 and POT1, as well as TRF1, as demonstrated by positive correlations (Figure 4). This is partially in line with data published by Braig et al. pointing at the important role of RAP1 in telomere maintenance in CML cells [31].

Our results demonstrated that the differences in telomere length between *BCR-ABL1*-positive cell lines can be explained only partially by telomerase activity (Figure 3). Another molecular mechanism, which may be responsible for observed variation in telomere lengthening is the alternative lengthening of telomere (ALT) phenomenon. The role of shelterin proteins in alternative lengthening of telomeres in chronic myeloid leukemia has not been studied in detail up to now, therefore we have decided to analyze co-localization of alternative lengthening of telomeres-associated promyelocytic leukemia (PML) nuclear bodies (APBs) with TRF2 commonly used as a marker of ALT. The APB contains telomeric DNA, extrachromosomal telomeric circles (t-circles), DNA repair proteins and the telomere repeat binding factors such as TRF2 [37,38,39]. Our immunofluorescence analysis showed that TRF2 was co-localized with PML only in K-562 cells (Figure 5). These results may suggest that telomere length in K562 despite activity of telomerase may be also associated with ALT phenomenon, which is confirmed by previous study showing that the t-circles, one of the ALT hallmarks, can be used to define ALT activation in CML patients [11]. Samassekou et al. have shown that in 27% CML-CP cells exhibited both high ALT activity and telomerase activities. As telomerase activity increases with disease progression, the dominating telomere maintenance mechanism might undergo transition from ALT to telomerase. In this context, both the consequence and the origin of telomere shortening are still a matter of debate. 

Telomere maintenance dysfunction in cancer cells is commonly associated with genomic instability [40]. We have used common markers to monitor the genomic instability, such as micronuclei, amount of DNA in Tail (comet assay), or recruitment of H2AX (Figure 5). In this study, we tested the hypothesis that upregulation of telomeric proteins may result in increased pool of telomere-unbound proteins as an adaptation to oxidative stress conditions. This may promote the inhibition of ATM/ATR and cause the attenuation of DNA damage response (DDR), e.g., limited recruitment of H2AX, which may lead to an increase in the global genomic instability and clonal selection (Figure 5). Surprisingly, we have found that level of micronuclei production (Figure 1) and number of double strand breaks as measured by comet assay (Figure 5) in cells without exogenic stress were not associated with *BCR-ABL1* copy number. However, upon etoposide treatment, the activation of ATM kinase in K562, LAMA-84 and MEG-2A cell lines was more pronounced than in HL-60 cells. Simultaneously the histone H2AX phosphorylation on Ser139 in response to DNA damage was less pronounced in K562 than in KU-812 and MOLM-1 (Figure 5). 

This suggests that BCR-ABL1 translocates to the nucleus and associates with ATM after DNA damage but does not affect ATM function. This above observation is agreed with previous report showing that BCR-ABL1 disrupts ATR-dependent signal transduction events but not ATM function [41]. Additionally, we have noted that the lower phosphorylation of the histone H2AX on Ser139 in response to DNA damage corresponded with higher TRF2 level in K562 (Figure 4 and Figure 5). This could be result of blunting of the DNA damage response in K562 cells when TRF2 is overexpressed [42]. Interestingly, the comparison analysis of the levels of RAP1 identified as a TRF2-interacting protein between cell line has not revealed any significant relationship with the comet DBS assay, the micronucleus counts, phosphorylation of the histone H2AX on Ser139 in response to DNA damage (Figure 1, Figure 4, and Figure 5). The LAMA-84 cell line that has the one of higher level of RAP1 expression has almost similar level of DNA damage (micronuclei and DSB) compared to KU-812 or MEG-A2, cell lines with the lowest level of RAP1. Similar observation was noted upon etoposide treatment (Figure 5). Moreover, it is noted that K562, the cell line with the highest level of RAP1 and other shelterin proteins, has not shown any significant changes in the levels of genomic instability compared to other CML cell lines upon etoposide treatment (Figure 5). So, our results have not confirmed previous observation e.g., in gastric cancer cells treated with etoposide and we cannot conclude that shelterin proteins play important role in etoposide-mediated DNA damage response in CML cells [43]. The correlational analysis between telomere length and RAP1 or TFR2 level in analyzed cell line has revealed that is not significant correlation between telomere length with RAP1 (r = 0.68) and TRF2 level (r = 0.47) respectively. On the other hand, RAP1 is a well-known factor of DNA damage response, not only in telomeric, but also non-telomeric regions, so it is possible that RAP1-mediated genome stability may be based on its other protective role not related to direct telomere function in CML cell lines [44]. It was shown that mammalian Rap1 could be important for hematopoietic stem cell survival or response to chemotherapy through e.g., interaction between XRCC4/DNA Ligase IV and DNA-PK [45]. So, we conclude that only losing of RAP1 or other shelterin proteins like TRF1 or TRF2 could decrease double-strand break repair, but overexpression of these proteins is not able to promote clastogenic effects. Similar effect we have already observed in human dermal fibroblasts, HeLa, ACHN, A549, and MCF7 cells as we have also detected the overexpression of TRF1 and TRF2 as a result of stress condition. Simultaneously, we have observed limited recruitment of 53BP1 without any telomere length changes [46]. Therefore, we supposed that upregulation of TRF proteins results in an increased pool of telomere unbound TRFs that regulate the expression of subtelomeric genes as part of cellular adaptive response, however, this phenomenon should be studied in detail in the future.

Blastic phase of CML, though rare in the era of TKI, remains incurable except patients in whom allogeneic hematopoietic stem cell transplantation was successful. Studies on molecular pathogenesis of BP-CML showed high level of genomic instability and additional genetic aberrations, independent of BCR-ABL1 activity [3] also in leukemic stem cells compartment [5,47]. While this knowledge helped in our understanding of the molecular mechanisms of drug resistance and/or progression, it did not translate yet into effective targeting and treatment of patients in acute phase of CML. Unraveling the mechanisms of telomere maintenance and the role of telomerase and shelterin complex in BCR-ABL1-mediated genomic instability may contribute to the development of new strategies preventing or counteracting resistance phenotype and malignant progression of the disease. Targeting telomerase in acute myeloid leukemia proved that strategy aiming at the aberrant regulation of telomere maintenance may help to eradicate leukemic stem cells [48]. 

In summary, this work focused on BP-CML cell lines shows a complex picture of the regulation of telomere length and expression of telomere-related genes in BP-CML cells supporting the crucial role of BCR-ABL1 kinase expression and activity in the maintenance of telomeres. Presented results may help to validate and properly interpret results obtained by many laboratories employing these widely used cellular in vitro models of CML. Additionally, our data can be applied in the search for new therapeutic opportunities not only for advanced CML, but also in other acute leukemias.

## Figures and Tables

**Table 1:** Mutational status of selected genes in *BCR/ABL1* positive cell lines and HL-60 as determined by targeted enrichment and next-generation sequencing. Analysis of *TERT* promoter hotspot mutations was confirmed by Sanger sequencing. CNV is additionally given for *TP53* and *CDKN2A*. ^#^ copy loss corresponds to the loss of one allele while deletion to loss of both alleles (in HL-60 cell line manual inspection in IGV software of *TP53* exons revealed exons 1, 5 and 6). * describes nonsense variant in protein coding.

	Gene		K562	MEG-A2	MOLM-1	LAMA-84	KU-812	HL-60
**Telomerase complex genes**	*TERT* promoter (C228T and C250T)		-	-	-	-	-	-
	*TERT*		p.R888Q	-	-	-	-	-
	*TERC*		-	-	-	-	-	-
	*DKC1*		-	-	-	-	-	-
**Shelterin and telomere maintenance associated genes**	*RAP1*		-	-	-	-	-	-
	*POT1*		-	-	-	-	-	-
	*ATRX*		-	-	-	-	-	-
**Cancer related genes, mutated in at least two cell lines**	*ASXL1*		p.Y591 *	-	-	p.G643_G644fs	p.R693 *	-
	*ATM*		-	p.A1812V	p.R924Q	p.Q1128R	-	-
	*TP53*	mutation	p.Q136_L137fs	p.R273H	p.R196 *	p.K319*	p.K132R	-
		CNV ^#^	copy loss	copy loss	copy loss	copy loss	-	deletion
	*CDKN2A*	mutation	-	-	-	-	-	p.R80 *
		CNV ^#^	deletion	deletion	-	deletion	-	copy loss

## Supplementary Materials

The following are available online at https://www.mdpi.com/2073-4425/11/10/1145/s1, Supplementary Tables file including Table S1: List of genes covered by NGS capture panels 1 and 2; Table S2: CNV calling results and Table S2 notes; Supplementary Figures file including Figure S1: Representative images of fluorescence in situ hybridization with BCR-ABL1 t(9;22) fusion probe, JAK2 (9p24) break probe and PDGFRB (5q32) break probe in leukemic cell line. Please note that selected the single nuclei from this panel is present in main text of manuscript. Images were taken using an Olympus BX61 fluorescent microscope with objective 40×; Figure S2: Representative images of nuclei of leukemic cell line after hybridization with telomeric—PNA. Please note that selected the single nuclei from this panel is present in main text of manuscript. Images were taken using an Olympus BX61 fluorescent microscope with objective 40×; Figure S3: TERT (c.2852G > A) mutation in K562 cell line. (a) NGS results visualized by IGV software. Total number of reads together with variant allele frequency (VAF) are shown; (b) Confirmation of detected variant by Sanger sequencing presented as a screenshot from the FinchTV. The changed nucleotide is shown in a box. Reference and mutated sequences are given at the top; Figure S4: C228T and C250T TERT promoter hot spots sequenced by Sanger sequencing presented as a screenshot from the FinchTV. The reference sequence together with mutated sequences are given at the top. Red arrows and green boxes indicate the locations of the most common hot spots C228T (right) and C250T (left); Figure S5: CNV calling results for K562 presented as a scatter plot; Figure S6: CNV calling results for LAMA-84 presented as a scatter plot; Figure S7: CNV calling results for MOLM-1 presented as a scatter plot; Figure S8: CNV calling results for MEG-A2 (re-centered) presented as a scatter plot; Figure S9: CNV calling results for KU-812 (re-centered) presented as a scatter plot; Figure S10: CNV calling results for HL-60 presented as a scatter plot; Figure S11: ALT phenomenon in BCR/ABL1-positive cell lines: Co-localization promyelocytic leukemia protein (PML) (green) and TRF2 (red) by immunofluorescence with anti-PML and anti-TRF2 antibodies. Nuclei were visualized by DAPI staining (blue). Images were taken using an Olympus BX61 fluorescent microscope with objective 20×. To analyze colocalization PML/TRF2, ImageJ software http://rsbweb.nih.gov/ij/ with JACoP plugin was used (Bolte & F. P. Cordelières, A guided tour into subcellular colocalization analysis in light microscopy, Journal of Microscopy, Volume 224, Issue 3: 213–232.). The Pearson’s coefficient was used to calculating a set of co-localization indicator.
