# Supplementary material for: Differential Regulation of Telomeric Complex by BCR-ABL1 Kinase in Human Cellular Models of Chronic Myeloid Leukemia—From Single Cell Analysis to Next-Generation Sequencing
Source: Genes (Basel). 2020 Sep 29;11(10):1145. doi: 10.3390/genes11101145 (PMC7601685; doi:10.3390/genes11101145)
Supplement: Supplementary file 1 [file genes-11-01145-s001.zip › supplementary files/Supplementary Figures-1.pdf]

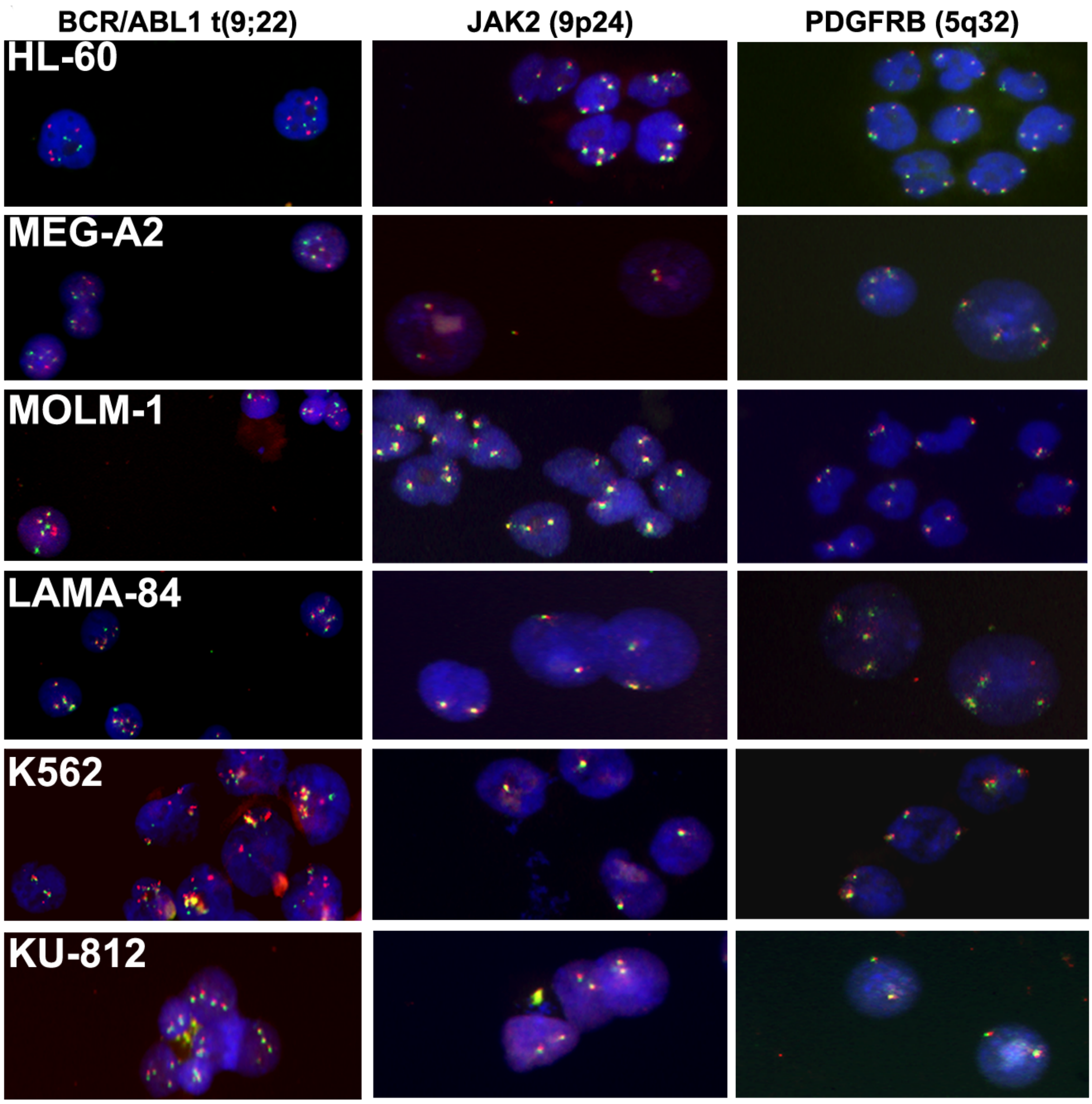

Figure S1: Representative images of fluorescence in situ hybridization with *BCR-ABL1* t(9;22) fusion probe, *JAK2* (9p24) break probe and *PDGFRB* (5q32) break probe in leukemic cell line. Please note that selected the single nuclei from this panel is present in main text of manuscript. Images were taken using an Olympus BX61 fluorescent microscope with objective 40×

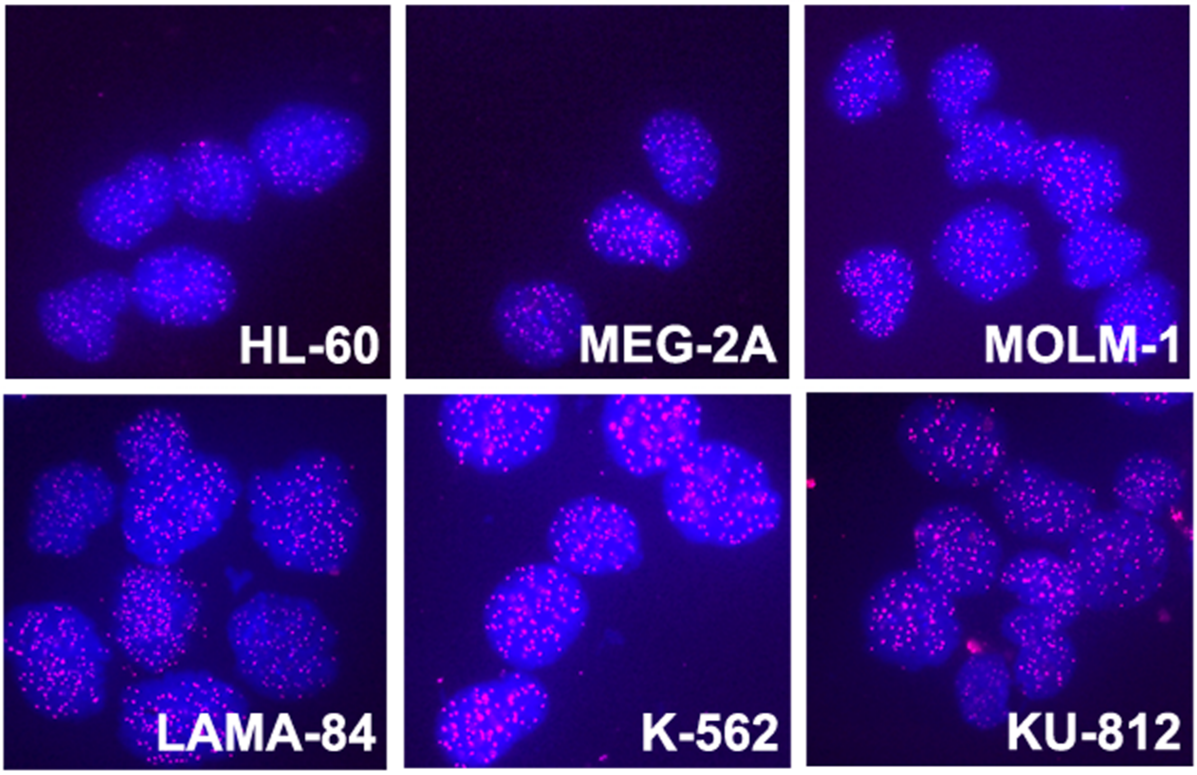

Figure S2: Representative images of nuclei of leukemic cell line after hybridization with telomeric – PNA. Please note that selected the single nuclei from this panel is present in main text of manuscript. Images were taken using an Olympus BX61 fluorescent microscope with objective 40×

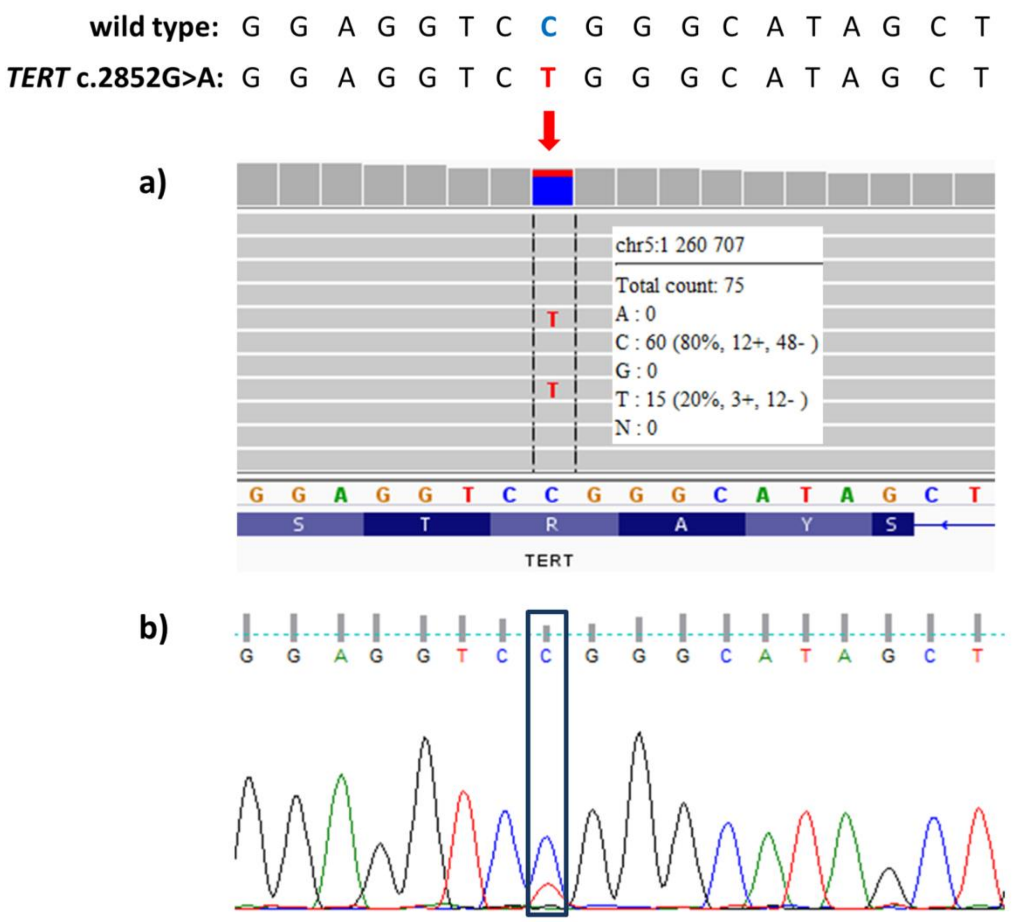

Figure S3: *TERT* (c.2852G>A) mutation in K562 cell line. (**a**) NGS results visualized by IGV software. Total number of reads together with variant allele frequency (VAF) are shown; (**b**) Confirmation of detected variant by Sanger sequencing presented as a screenshot from the FinchTV. The changed nucleotide is shown in a box. Reference and mutated sequences are given at the top.

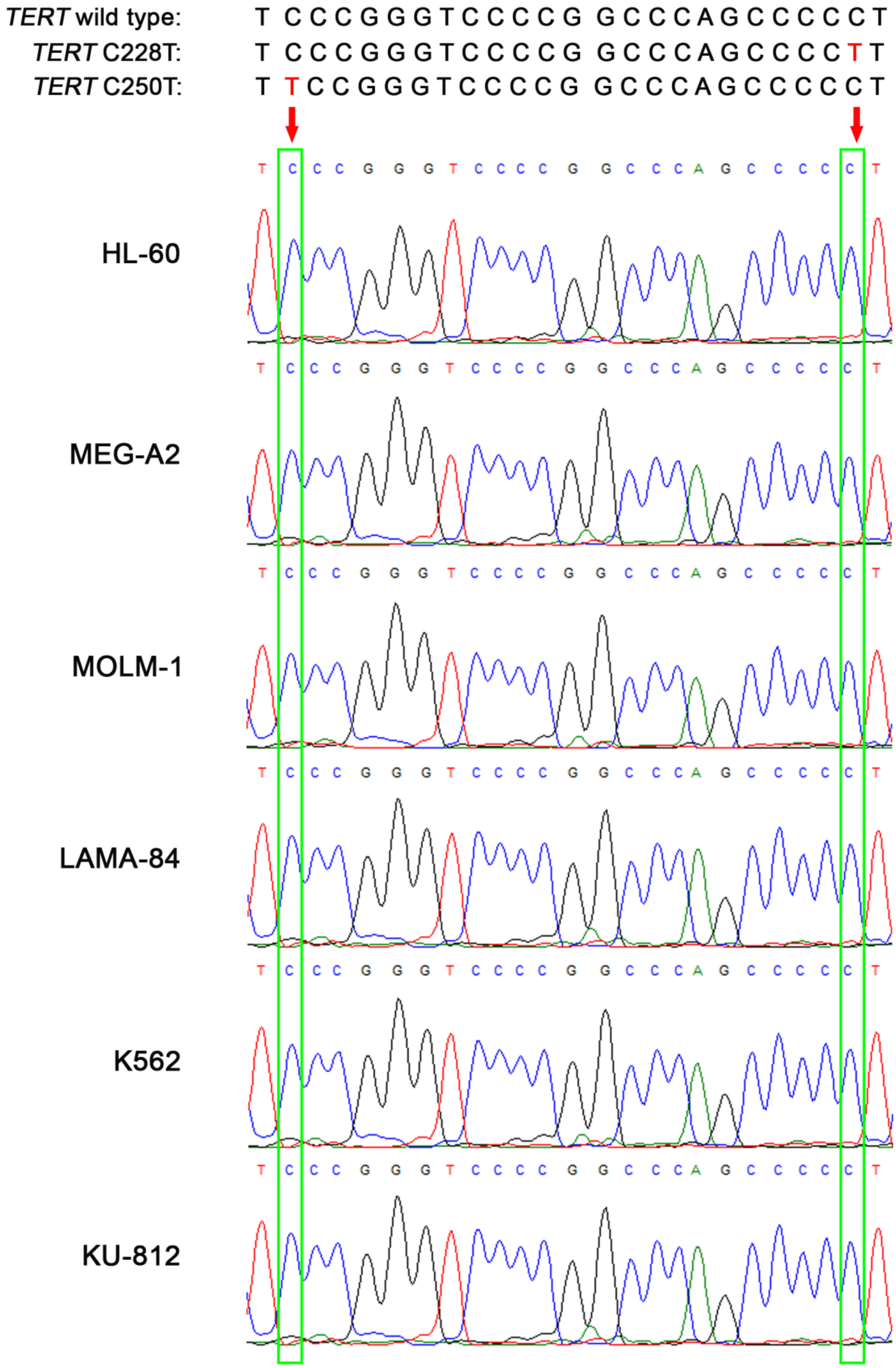

Figure S4: C228T and C250T *TERT* promoter hot spots sequenced by Sanger sequencing presented as a screenshot from the FinchTV. The reference sequence together with mutated sequences are given at the top. Red arrows and green boxes indicate the locations of the most common hot spots C228T (right) and C250T (left).

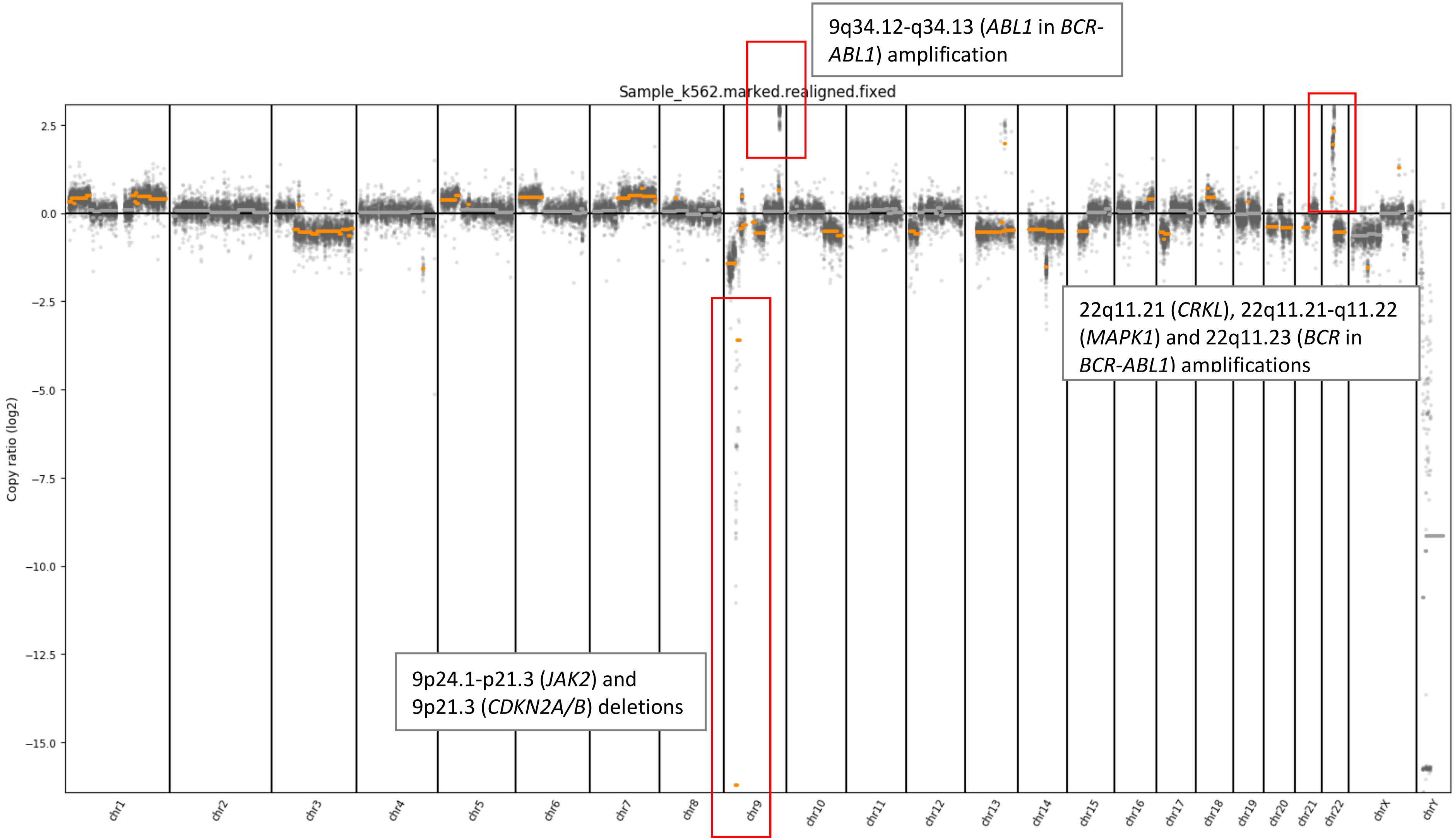

Figure S5: CNV calling results for K562 presented as a scatter plot.

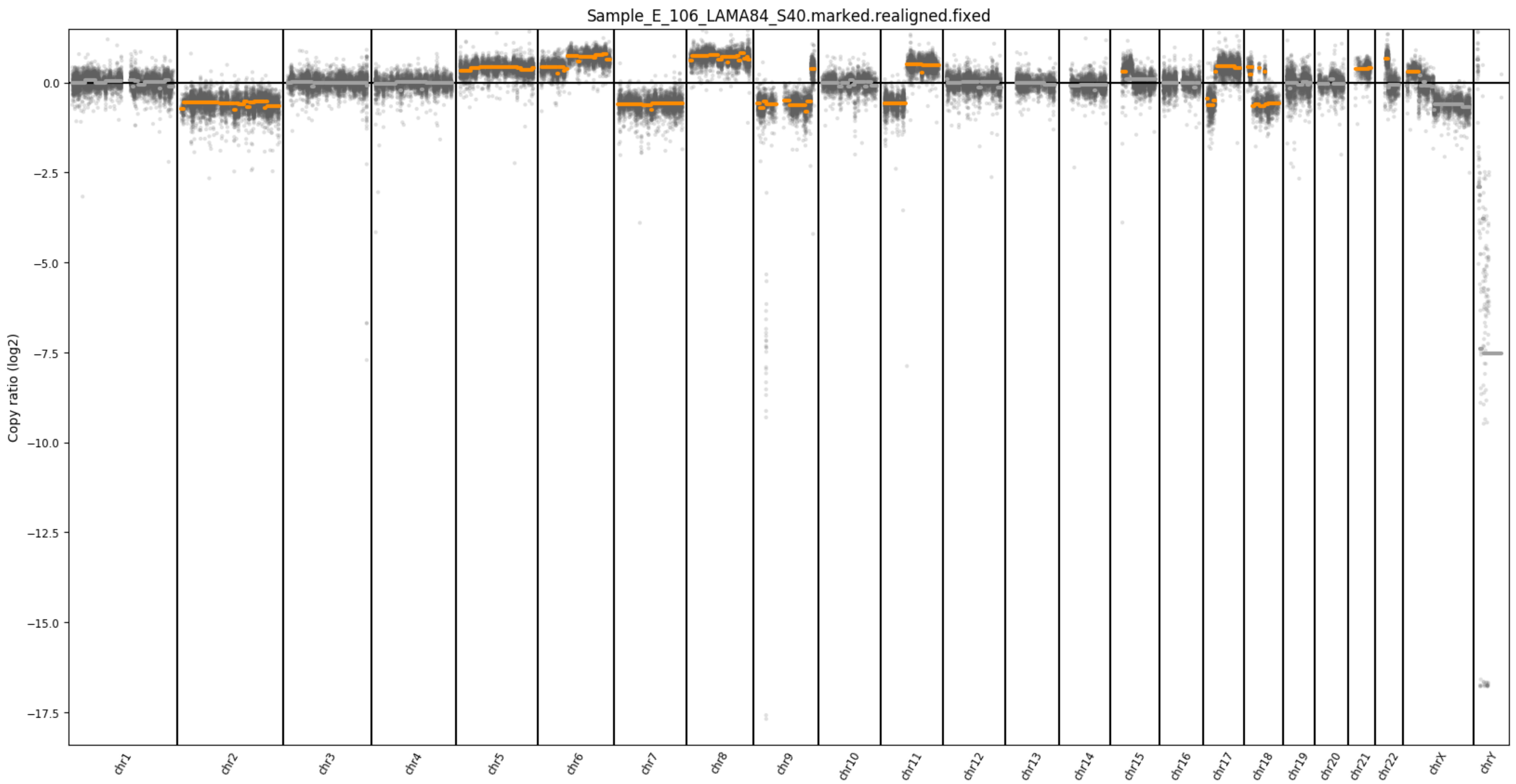

Figure S6: CNV calling results for LAMA-84 presented as a scatter plot.

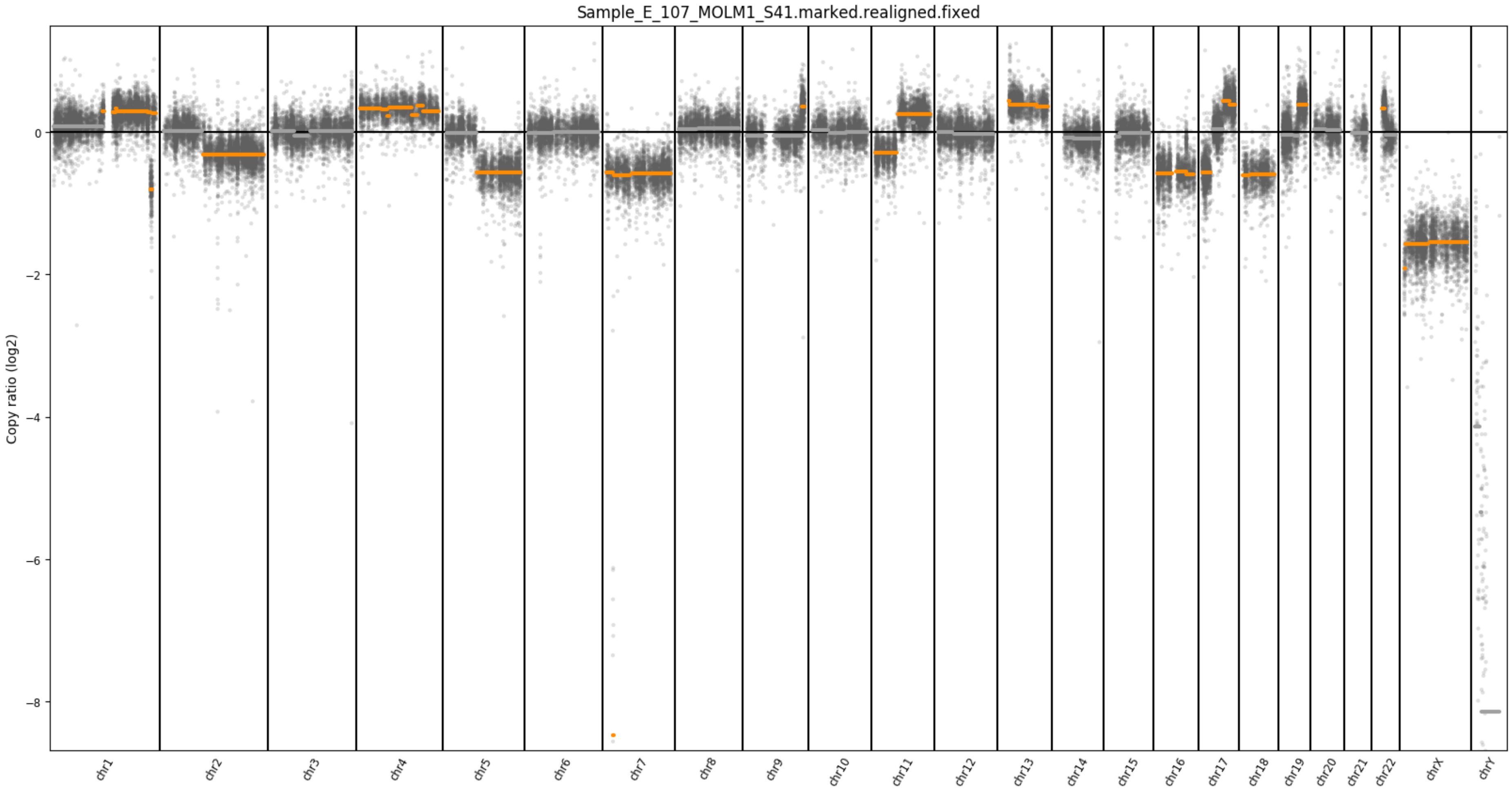

Figure S7: CNV calling results for MOLM-1 presented as a scatter plot.

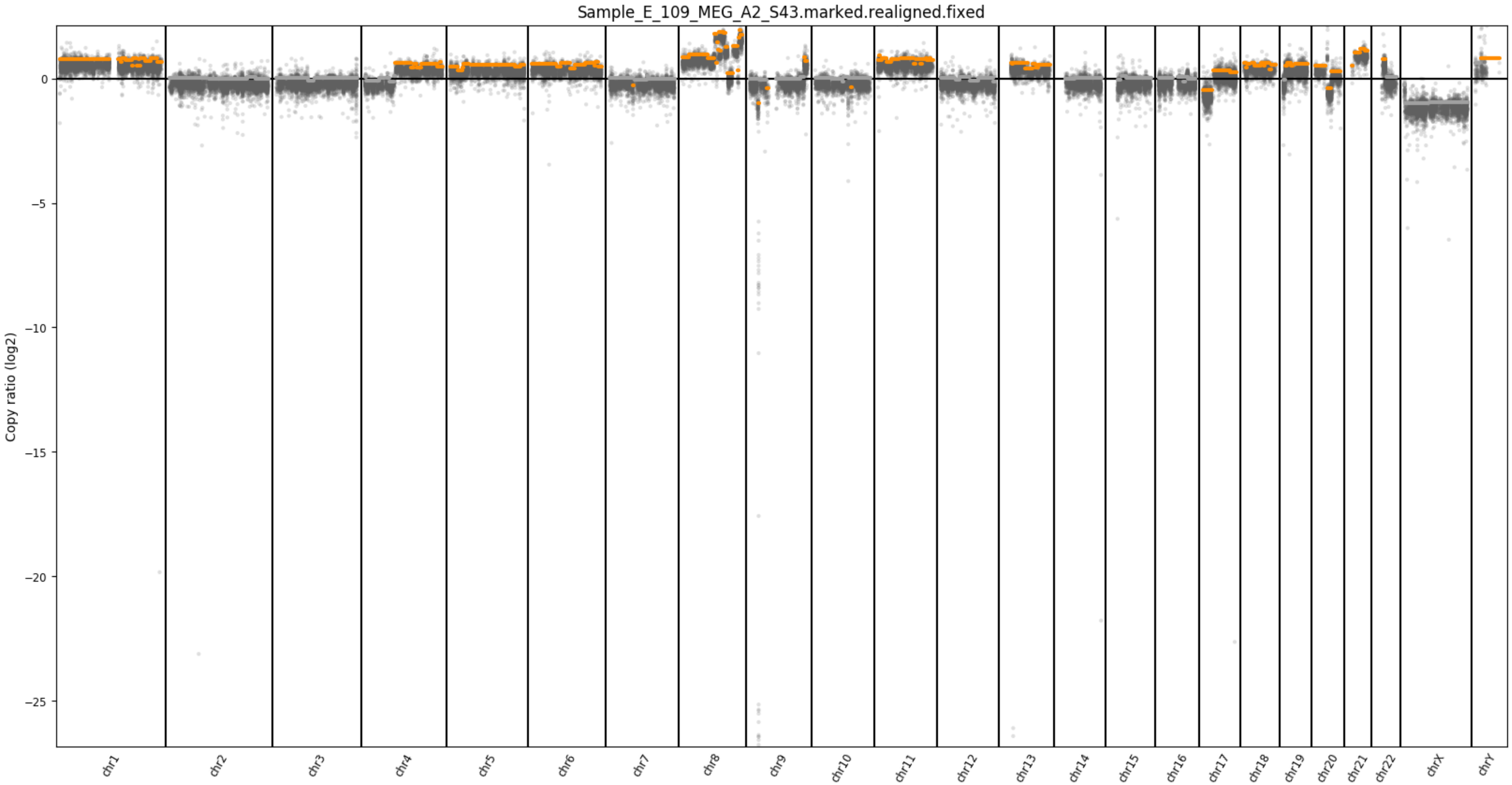

Figure S8: CNV calling results for MEG-A2 (re-centered) presented as a scatter plot.

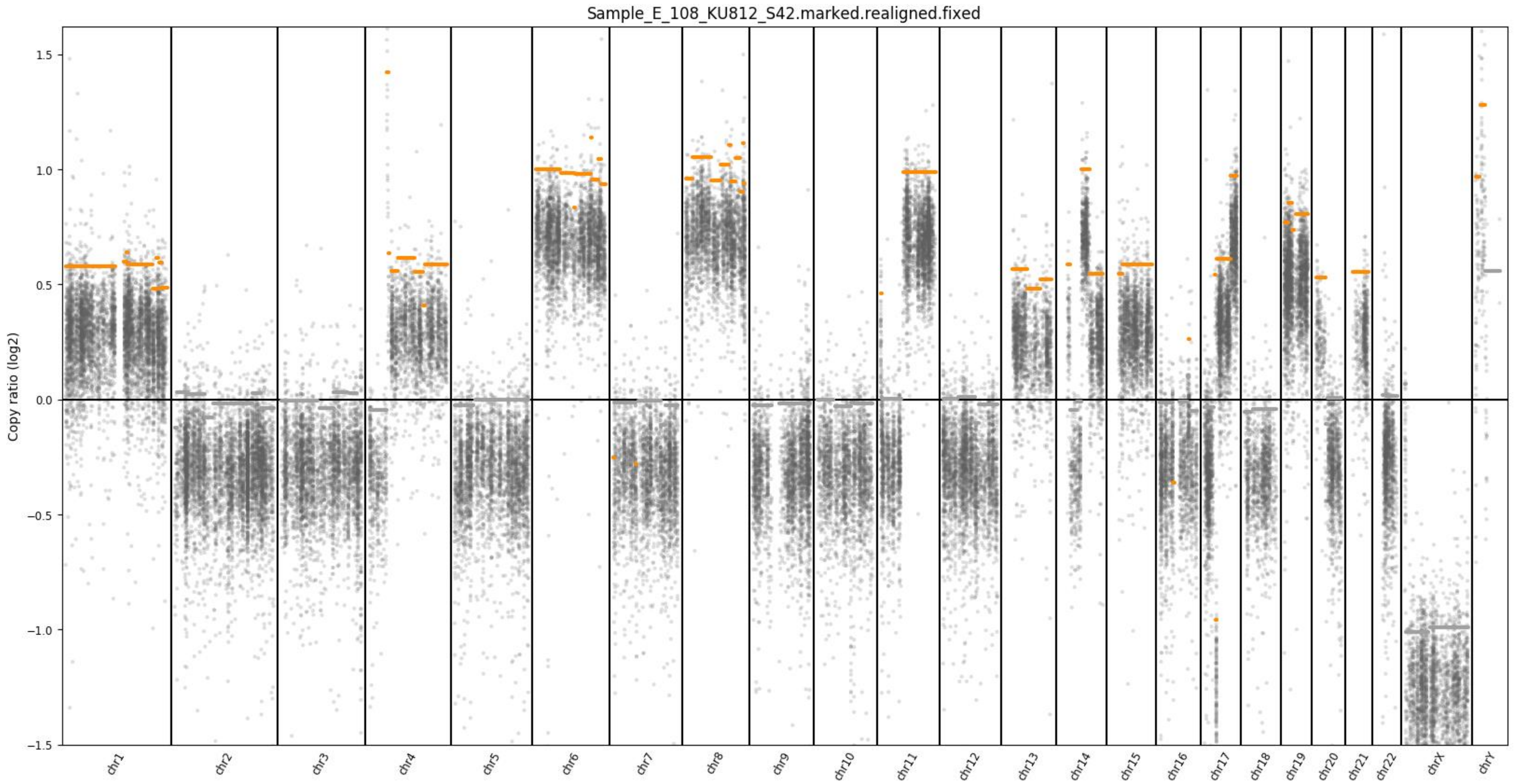

Figure S9: CNV calling results for KU-812 (re-centered) presented as a scatter plot.

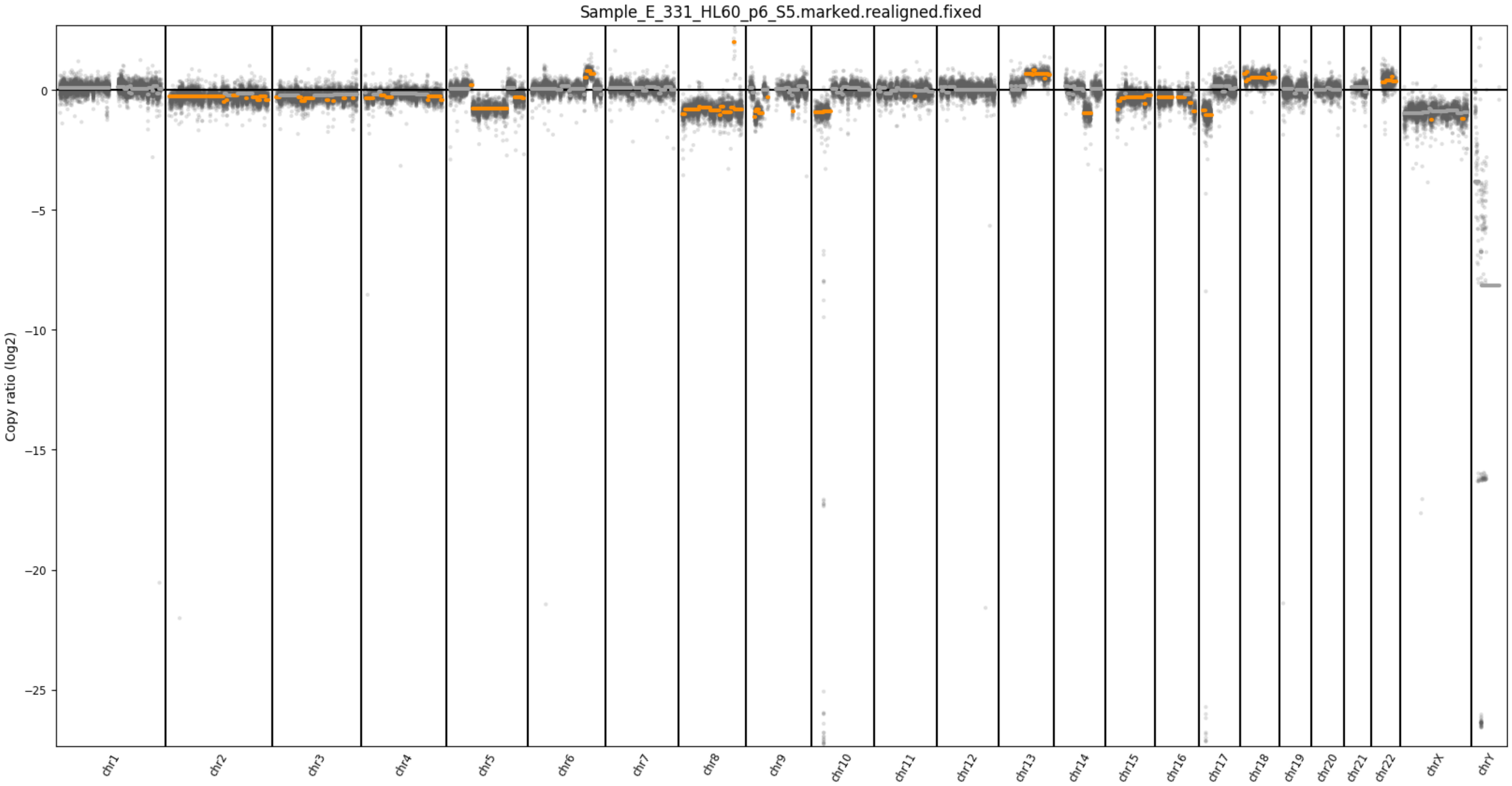

Figure S10: CNV calling results for HL-60 presented as a scatter plot.

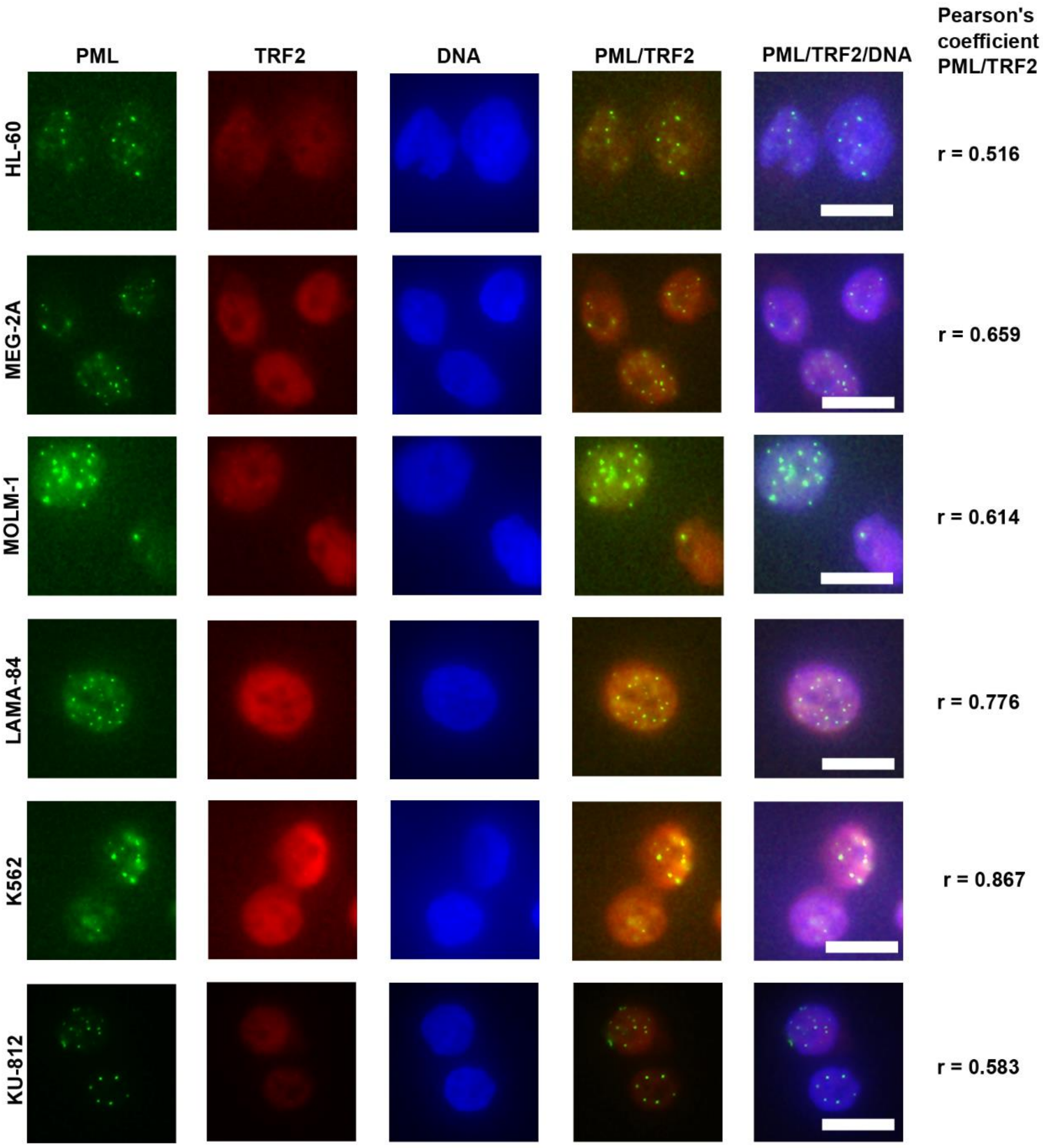

Figure S11: ALT phenomenon in *BCR/ABL1*-positive cell lines: Co-localization promyelocytic leukemia protein (PML) (green) and TRF2 (red) by immunofluorescence with anti-PML and anti-TRF2 antibodies. Nuclei were visualized by DAPI staining (blue). Images were taken using an Olympus BX61 fluorescent microscope with objective 20×. To analyze colocalization PML/TRF2, ImageJ software http://rsbweb.nih.gov/ij/ with JACoP plugin was used (Bolte & F. P. Cordelières, A guided tour into subcellular colocalization analysis in light microscopy, Journal of Microscopy, Volume 224, Issue 3: 213-232.). The Pearson's coefficient was used to calculating a set of co-localization indicator.
